# Legacy of the Tehran Obesity Treatment Study: Findings from 10 Years Bariatric Surgery Survey

**DOI:** 10.5812/ijem-151608

**Published:** 2024-10-29

**Authors:** Behnaz Abiri, Minoo Heidari Almasi, Farhad Hosseinpanah, Danial Molavizadeh, Alireza Khalaj, Maryam Mahdavi, Majid Valizadeh, Maryam Barzin

**Affiliations:** 1 Obesity Research Center, Research Institute for Endocrine Sciences, Shahid Beheshti University of Medical Sciences, Tehran, Iran; 2 School of Medicine, Kashan University of Medical Sciences, Kashan, Iran; 3 Department of Surgery, Tehran Obesity Treatment Center, Faculty of Medicine, Shahed University, Tehran, Iran

**Keywords:** Tehran Obesity Treatment Study, TOTS, Bariatric Surgery, Severe Obesity, Sleeve Gastrectomy, Gastric Bypass

## Abstract

**Context:**

This paper aims to review the findings of the Tehran Obesity Treatment Study (TOTS) on obesity and bariatric surgery (BS).

**Evidence Acquisition:**

The objective of this review is to assess all aspects of BS in individuals with severe obesity, focusing on research conducted within the TOTS framework.

**Results and Conclusions:**

The TOTS studies have produced significant national-level findings, highlighting critical issues related to the effectiveness and outcomes of bariatric procedures, the importance of comprehensive nutritional management, and the complications associated with these interventions in this population.

## 1. Context

The Tehran Obesity Treatment Study (TOTS) is an ongoing, single-center, prospective research project that began in March 2013. The TOTS involves enrolling patients for bariatric procedures based on a personalized clinical decision plan, structured into four phases: Preoperative evaluation, surgery, short-term follow-up, and long-term follow-up (1). The primary objectives of the study are to identify perioperative challenges in morbidly obese patients, compare the effectiveness of various bariatric surgical techniques on obesity-related comorbidities by evaluating anthropometric indices, biochemical measurements, and nutritional status, and assess these parameters before and after bariatric surgery over an extended period.

The study includes various bariatric procedures, such as Roux-en-Y gastric bypass (RYGB), adjustable gastric banding (AGB), sleeve gastrectomy (SG), and one anastomosis gastric bypass (OAGB). A comprehensive measurement plan has been developed to document all aspects of the surgeries, from preoperative to postoperative phases. All procedures are performed by a single surgical team under general anesthesia. A digital database was established to ensure accurate data collection. Data collectors, including study investigators and surgeons, received training and certification in the study protocols. Quality control procedures, including regular communication and visits between surgeons and clinical center staff, were implemented to ensure thorough and precise data collection. Investigators used validated and standardized tools to assess both objective and subjective measures (2).

To date, a total of 7355 procedures have been performed, including 272 revisional surgeries. Among these, 7083 surgeries included 4834 SG (65.1%), 1950 OAGB (27.2%), 150 RYGB (2%), and 149 other techniques (2%). The follow-up rates for 12, 24, 36, 48, 60, and 72 months after surgery were 86.8%, 86.1%, 66.3%, 63.3%, 50.1%, and 34.2%, respectively.

In this review, we aimed to examine all research studies conducted on bariatric surgery for individuals with severe obesity within the TOTS framework, as outlined in our original objectives.

## 2. Evidence Acquisition

To identify all available publications related to the TOTS, we conducted a comprehensive literature search across multiple databases, including PubMed, Scopus, and Web of Science. The search utilized a combination of keywords and Medical Subject Headings (MeSH) terms such as "bariatric surgery," "obesity," "Tehran Obesity Treatment Study," and "weight loss." After removing duplicates, the remaining articles were screened for relevance based on titles and abstracts. This was followed by a full-text assessment using predefined inclusion criteria that focused on adult participants undergoing bariatric surgery within the TOTS framework. This systematic approach ensured a thorough overview of the existing literature on bariatric surgery outcomes for individuals with severe obesity.

## 3. Results

### 3.1. Effectiveness for Weight Loss

Effective weight loss and its maintenance are the most important outcomes after bariatric surgery (BS). In four articles from the TOTS study, we examined the effectiveness of weight loss across different types of BS by evaluating total weight loss (TWL) and excess weight loss (EWL). Our early study demonstrated the effectiveness and safety of SG compared to gastric bypass (GB) over a two-year follow-up period. The EWL% values were 61.9 ± 15.7, 74.8 ± 19.1, and 75.0 ± 21.9 for participants who underwent SG, and 62.7 ± 15.3, 77.5 ± 18.4, and 80.1 ± 20.8 for those in the GB group at the 6-, 12-, and 24-month follow-up periods, respectively. Excess WL% was significantly greater in the GB group at 12 and 24 months (P = 0.002) (3). 

**Figure 1.**
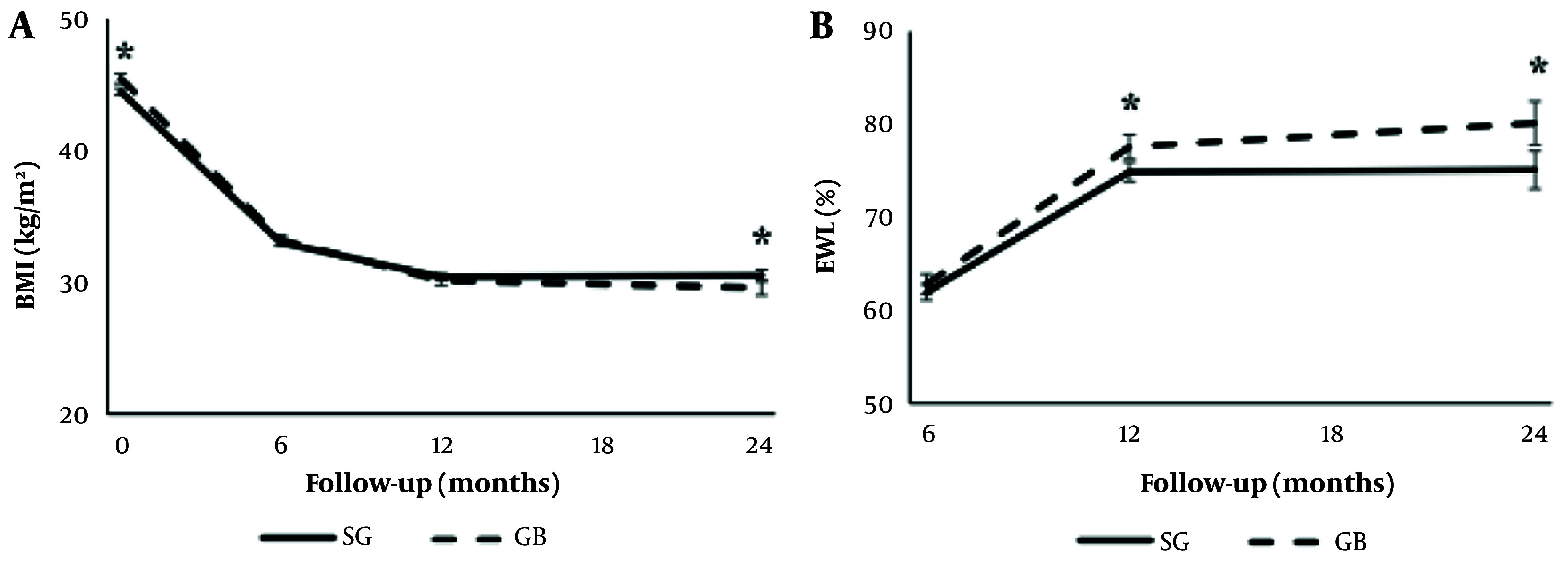
The anthropometric and body composition indices over time: A, body mass index (BMI); B, excess weight loss (EWL) (%). SG, sleeve gastrectomy; GB, gastric bypass. * P-value < 0.05.

Three-year follow-ups further revealed that TWL% and EWL% were 28.4 ± 9.0 and 33.8 ± 9.8, and 67.0 ± 22.4 and 76.4 ± 22.3, in the GB and SG groups, respectively (P between < 0.05) (Figure 1) (4).

The International Federation for the Surgery of Obesity and Metabolic Disorders (IFSO) has highlighted insufficient weight loss (IWL) and weight recurrence (WR) as primary concerns following bariatric surgeries. In a study involving 1,939 participants who underwent bariatric surgery (1,295 SG and 644 GB), participants were followed for 72 months to identify predictors of WR. The study utilized five definitions to determine WR: (1) An increase of ≥ 10 kg in weight, (2) a percentage weight increase ≥ 15% of nadir weight, (3) an increase in maximum weight loss ≥ 20%, (4) [100 × (post-nadir weight - nadir weight)]/(Pre-surgery weight - nadir weight), and (5) an increase of > 25% in EWL from nadir. Depending on the definition applied, the prevalence of WR varied significantly, ranging from 13.5% to 35.5%. 

**Figure 2.**
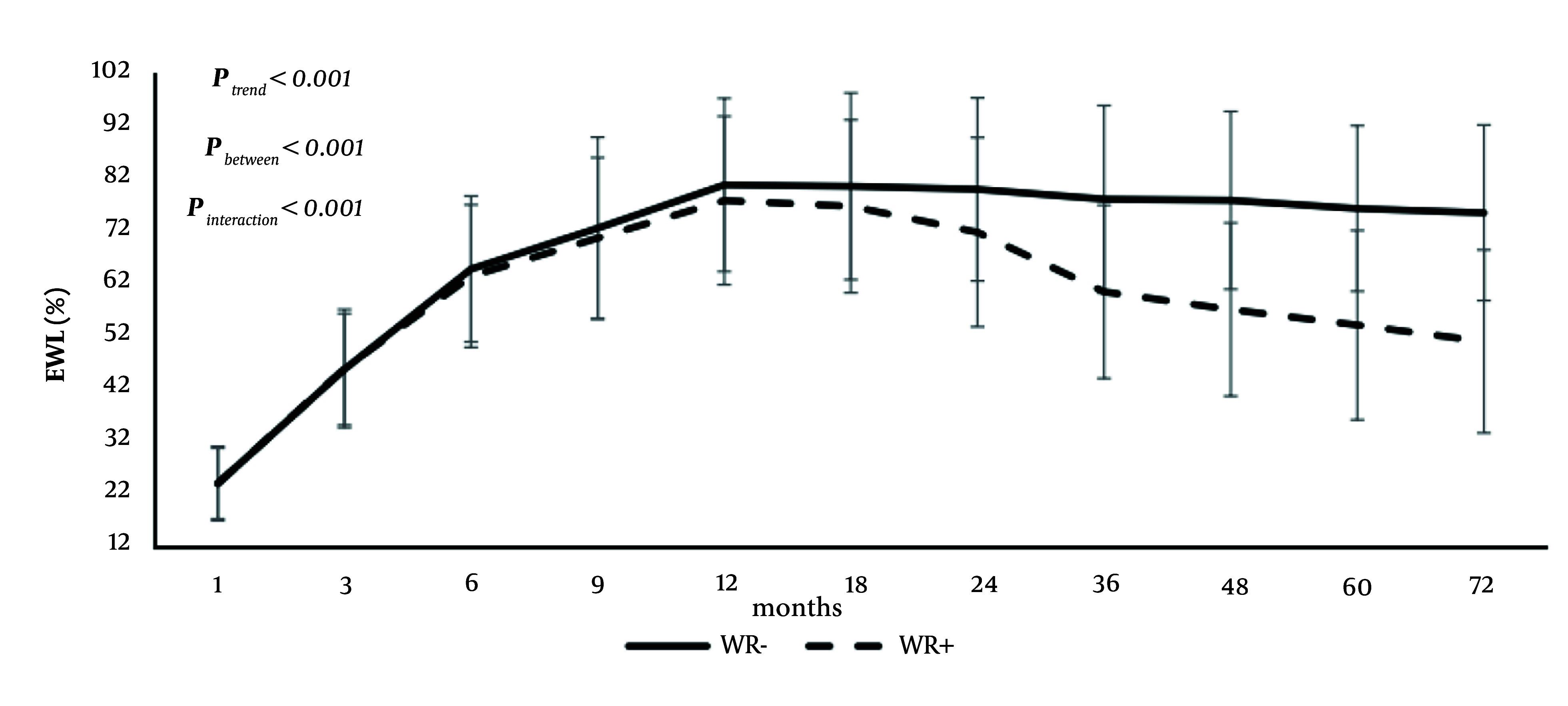
The trends of excess weight loss in the two study groups (i.e., weight regain and non-weight regain). Weight recurrence was defined based on maximum weight loss percentage ≥ 20%, and the patients were followed up for 72 months on average. WR, weight recurrence; EWL, excess weight loss.

The study found that WR tended to occur around 24 months post-surgery, and specific age and BMI cutoff points of 38 years and 45 kg/m² were identified as predictors of WR. The analysis revealed that SG (across all five definitions), a high preoperative BMI (in three of the five definitions), and younger age (in four of the five definitions) were the most significant risk factors for WR. Additionally, fat-free mass percentage (FFM%) emerged as a risk factor in two definitions, whereas hypertension and female gender were each identified as independent risk factors in one definition. Furthermore, the 72-month follow-up study demonstrated that participants without WR achieved a significantly greater EWL% compared to those with WR (Figure 2) (5).

In our unpublished data on IWL, we found that 8% of participants experienced insufficient weight loss. Insufficient weight loss was defined as achieving less than 50% of EWL over a median follow-up period of 18 months. Predictors of IWL, all with P-values < 0.05, included older age (95% CI: 1.501 - 6.677), higher baseline BMI (95% CI: 1.905 - 11.151), type 2 diabetes mellitus (95% CI: 1.063 - 2.477), and undergoing SG (95% CI: 2.531 - 7.554).

### 3.2. Effects on Body Composition

**Figure 3.**
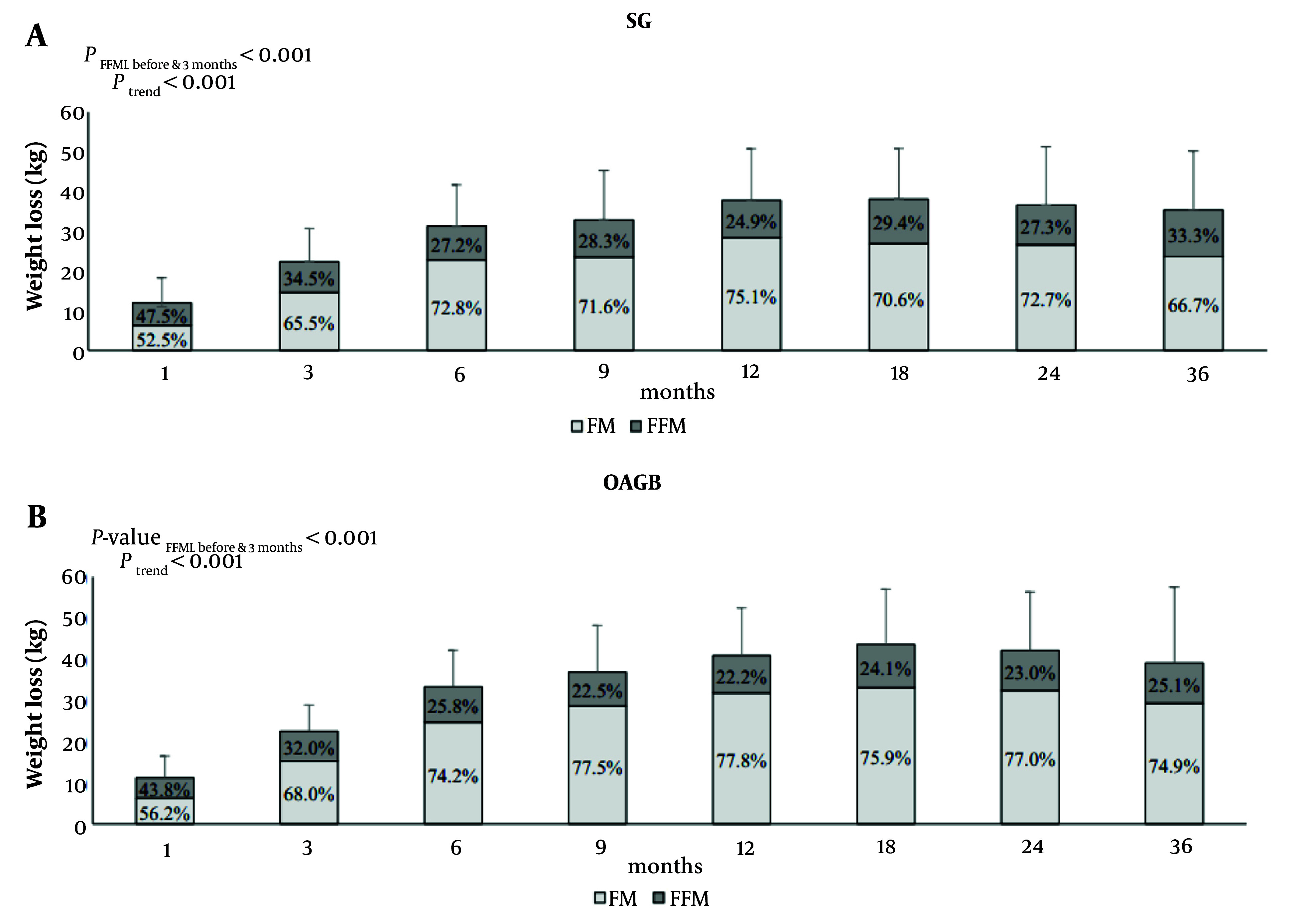
Weight loss with respect to preoperative weight and its proportion to fat mass (FM) loss and fat-free mass (FFM) loss in the sleeve gastrectomy (SG) (A); and one anastomosis gastric bypass (OAGB) (B) groups. P-value fat-free mass loss (FFML) before 3 months between surgery groups = 0.001; P-value FFML after 3 months between surgery groups = 0.697

One of our primary challenges lies in recognizing that bariatric surgery impacts not only fat mass (FM) but also fat-free mass (FFM), particularly among elderly adults at risk of sarcopenia. According to TOTS data reports on body composition changes post-SG and OAGB up to three years, the findings revealed significant decreases in weight, BMI, TWL%, FFM, FM, and fat-free mass loss/weight loss (FFML/WL%), alongside an increase in EWL% in both groups (Ptrend < 0.001). Furthermore, the OAGB group exhibited enhanced preservation of FFM and superior loss of FM over time compared to the SG group. Notably, FFML during the initial three months was more pronounced in the SG group than in the OAGB group. Determinants of FFML included female gender, higher preoperative BMI, and the SG surgical technique (Figure 3) (4).

### 3.3. Sub-groups Analysis

In six TOTS publications, we examined the effectiveness of BS in separate studies focusing on subgroups, including metabolically healthy severely obese individuals (MHSO), super obese individuals, older adults, adolescent populations, sex disparities, and chronic kidney disease (CKD). Metabolically healthy severely obese individuals represent a subgroup in which the role of BS, such as its effectiveness in weight loss and comorbidity reduction, remains unclear. In our reports comparing the effects of BS on body composition changes in MHSO and metabolically unhealthy severely obese (MUSO) patients, the MHSO subgroup exhibited significant reductions in BMI, WC, SBP, DBP, FPG, and TG, alongside an elevation in EWL% and HDL-C over a two-year postoperative period. While changes in BMI, EWL%, and WC were greater in MHSO subjects, changes in DBP, SBP, FPG, TG, and HDL-C were more pronounced in MUSO participants. Although the MHSO phenotype showed a relatively lower decrease in FFML/WL% and a more favorable increase in EWL and TWL at specific time points, no clinically significant disparities in body composition changes were observed between the groups throughout the 36-month period (6).

In our cohort study of 557 patients with super obesity, we observed that patients undergoing OAGB experienced more significant weight loss compared to those undergoing SG during the three years of follow-up. The TWL was 36.5% versus 33.2% (P < 0.001), respectively. Hypertension, dyslipidemia, and diabetes mellitus remission rates were similar after both bariatric techniques. Despite OAGB offering greater mid-term weight loss, SG remains the preferred option for patients with super obesity due to its comparable metabolic benefits and safer surgical profile (7).

Fifty-six patients aged 60 to 76 who underwent either SG or GB (RYGB and OAGB) were matched with 112 younger controls, aged 18 to 60, based on sex, preoperative BMI, and type of surgery. Surgical complications were comparable between the two groups. Postoperatively, improvements in diabetes mellitus (DM) and dyslipidemia were similar between the groups. Although the rates of hypertension (HTN) improvement were identical, HTN remission was more frequent in the control group. The control group demonstrated greater changes in weight, BMI, FFM, lean mass, EWL%, and TWL% compared to the older group. However, changes in FM and FM% were comparable between the two groups. These findings indicate that BS is a safe and effective approach for managing obesity-related comorbidities in older adults, with surgical complication rates similar to those in younger controls (8).

A study involving 118 adolescents aged 11 to 18 as the case group and 236 young adults aged 19 to 29 as the control group assessed the outcomes of BS (SG or GB). Both groups were matched based on sex, preoperative BMI, and type of surgery. Surgical outcomes were evaluated one-year post-surgery. The study revealed comparable average reductions in BMI and EWL% between the groups. Both groups also experienced similar reductions in cardiovascular risk factors and remission rates for HTN, DM, and dyslipidemia. However, young adults showed a more significant increase in hemoglobin levels and copper deficiency, whereas adolescents demonstrated a greater increase in ferritin deficiency. The study concluded that BS is a safe and effective approach for weight loss, managing obesity-related comorbidities, and improving cardiovascular risk factors in adolescents, yielding results comparable to those observed in young adults (9).

Our study, which matched 707 men with 707 women based on age, preoperative BMI, and type of surgery, evaluated and compared the remission of comorbidities, weight loss, and complications associated with bariatric surgery. We found no significant differences between men and women regarding intraoperative bleeding, operation duration, or hospital stay length. Data analysis showed comparable TWL%, BMI reduction, and percentage of excess BMI loss at 12, 24, and 36 months postoperatively between the sexes. Furthermore, there were no significant differences in the remission rates of hypertension, diabetes mellitus, and dyslipidemia at 12 months (10). 

Another TOTS study evaluating BMI, TWL%, EWL%, and IWL based on preoperative eGFR categories (30 - 59, 60 - 89, 90 - 124, and ≥ 125 mL/min) revealed that patients with higher preoperative eGFR achieved more substantial weight loss. However, eGFR itself does not appear to independently influence weight loss outcomes (11).

### 3.4. Improvement of Comorbidities

Although weight loss is one of the primary goals of bariatric surgeries, the improvement or remission of comorbidities is equally significant. Therefore, we examined the remission of comorbidities over one to five years of follow-up in five TOTS publications. We compared 612 morbidly obese individuals who underwent bariatric surgery in TOTS with 593 individuals from the Tehran glucose and lipid study (TLGS) control group, who had not undergone surgical intervention, in an observational cohort study. The results showed that the development of DM was 2.9% in the bariatric surgery group compared to 15% in the control (lifestyle modification) group. New-onset hypertension and dyslipidemia were also less frequent in the bariatric group than in the control group (4 [1.8%] vs. 70 [20.4%] and 33 [14.3%] vs. 93 [31.5%], respectively). The relative risk reductions associated with bariatric surgery for the onset of DM, HTN, and dyslipidemia were 94%, 93%, and 55%, respectively, even after accounting for the poorer metabolic profile in the surgery group (12).

In our 12-month follow-up comparing SG and GB regarding weight loss, glycemic control, and metabolic syndrome (MetS) resolution, the prevalence of MetS decreased from 64% to 10% in the GB group and from 60% to 16% in the SG group, with no statistically significant difference between the groups (13).

Our two-year follow-up study showed that the remission rate of DM (defined as HbA1c < 6.5% and FBG < 126 mg/dL without antidiabetic medications) was comparable between the SG and GB techniques, with 53.3% in the SG group and 63.8% in the GB group (3). The remission rate for HTN (defined as SBP < 140 mmHg and DBP < 90 mmHg without antihypertensive medications) was greater in the GB group (54.7% vs. 39.1%) (3). The cumulative incidence of HTN remission and relapse was 83.9% (95% CI: 81.6 - 86.5) and 31.4% (95% CI: 25.6 - 38.2), respectively, with no significant difference between SG and GB. Our findings indicated that participants who were younger and used fewer antihypertensive medications before surgery benefited the most in terms of HTN remission following bariatric surgery (14).

We compared SG (n = 682) and GB (n = 355) participants in terms of alanine transaminase (ALT), aspartate transaminase (AST), and alkaline phosphatase (ALP). Both bariatric surgery methods demonstrated improvements in liver function parameters; however, SG showed more favorable trends compared to GB within the first year (15).

Additionally, we assessed non-alcoholic fatty liver disease (NAFLD) and liver fibrosis in bariatric patients prior to surgery using ultrasound, the NAFLD fibrosis score (NFS), and the fibrosis index-4 (FIB-4) in a cohort of 1,944 patients. A subset of 73 patients also underwent left liver lobe needle biopsies during surgery. The investigation revealed that approximately 70% of bariatric surgery candidates had NAFLD, with 10% showing signs of steatohepatitis in biopsy samples. Older age and higher transaminase levels were associated with higher NAFLD activity scores. Among the patients, 23.3% had fibrosis, predominantly at stage F1. Ultrasound identified steatosis in 76.8% of the patients, with two-thirds exhibiting grade I to II fatty liver. This study confirmed that while NAFLD is highly prevalent in severely obese patients, liver fibrosis is generally low-grade and uncommon (16).

### 3.5. Nutrition Insights from the Tehran Obesity Treatment Study

The TOTS has provided valuable insights into the nutritional challenges faced by individuals undergoing BS. Through a series of nine studies, ranging from pre-operative assessments to long-term dietary patterns post-surgery, significant findings have emerged, highlighting the importance of comprehensive nutritional management in this population.

The first study within TOTS focused on assessing the prevalence of micronutrient deficiencies among morbidly obese individuals scheduled for BS. Alarmingly, significant deficiencies in essential nutrients such as vitamin D (53.6%), vitamin B12 (34.4%), iron (10.2%), and low levels of hemoglobin (16.6%) were identified. Over half of the participants exhibited low levels of 25(OH)D, and approximately one-third experienced vitamin B12 deficiency (17).

Following BS, a study evaluated nutrient adequacy and deficiencies among patients one year post-operation, revealing inadequate intake of key nutrients like vitamin B12 (30%), ferritin (19%), and vitamin D (16.2%) in both SG and GB patients. In addition, insufficient protein intake (>80%) and total fat intake (>70%) were reported (18). Another study examined dietary nutrient intake six and 12 months after BS, identifying significant decreases in protein intake and inadequate intake of micronutrients such as biotin, fat-soluble vitamins, pantothenic acid, zinc, and potassium. None of the participants achieved adequate intake levels for fat-soluble vitamins, biotin, pantothenic acid, potassium, or zinc. Moreover, less than 10% of the participants maintained adequate intake of folate, magnesium, manganese, and calcium during both post-bariatric surgery intervals (19).

Additionally, in a prospective study with three years of follow-up, incident anemia was compared among 2,618 participants who underwent SG and OAGB. After the third year, although iron and vitamin B12 deficiencies remained comparable between the two surgery groups, the incidence of anemia was significantly higher among OAGB patients (56.5%) compared to those who underwent SG (24.3%) (20).

Another investigation in TOTS explored the metabolic relationship between parathyroid hormone (PTH) and 25-hydroxyvitamin D (25(OH) vitamin D) following BS. Given the absorption disturbances following BS, vitamin D insufficiency could lead to secondary hyperparathyroidism (SHPT). An observational prospective study involving 517 participants estimated a 12.6% overall prevalence of SHPT, which was more prominent among OAGB participants compared to the SG group (17.1% and 9.9%, P = 0.017) (21). However, 25(OH) vitamin D and calcium concentrations remained comparable. In addition, elevated preoperative iPTH levels (even within the normal range) and lower 1-year weight loss were identified as the main predictors of this complication. Findings suggested a shift in the threshold 25(OH) vitamin D concentration required to suppress iPTH post-surgery, emphasizing the need for tailored interventions to manage vitamin D metabolism and related hormonal responses in severely obese individuals undergoing surgery.

Moreover, TOTS investigated diet quality and anthropometric outcomes post-bariatric surgery, revealing suboptimal dietary patterns across food groups (especially for fruits, vegetables, oils, whole grains, and protein foods) and varied BMI trends over three years post-surgery (22). Similarly, the seventh study (22) focused on dietary intake and eating behaviors among patients at different intervals following SG. Participants were divided into three groups based on the time elapsed since SG and the timing of eating data collection: Group 1 (1 - 2 years), group 2 (2 - 3 years), and group 3 (3 - 5 years). Patients who were 3 - 5 years post-SG had a higher intake of energy and carbohydrates than those who were 1 - 2 years post-surgery. Additionally, the protein and overall macronutrient quality, as well as overall diet quality, decreased over time following surgery. The results indicated changes in energy and macronutrient intake over time, although eating behavior scores did not differ between groups (23).

Another study investigated the association between dietary quantity and quality with TWL and fat-free mass loss (FFML) at mid-term-post SG. This cross-sectional study involved 146 adults within 2 to 4 years post-SG. Patients who consumed more energy from carbohydrates had a lower percentage of TWL% 2 - 4 years after SG. Additionally, substituting an isocaloric amount of carbohydrates or fats with protein was linked to a reduced likelihood of non-response to SG. There was also a positive correlation between fat intake and FFML%. Furthermore, individuals who consumed more fiber exhibited a lower percentage of FFML% and reduced odds of excessive fat-free mass (FFM) loss (24).

Additionally, researchers from the TOTS explored the relationship between eating habits and the quantity and quality of diet in the mid-term following SG. This cross-sectional study also included 146 adults who were 2 to 4 years post-SG. Individuals with high emotional eating tendencies were observed to consume more energy and have a higher Healthy Plate Protein Quality Index (HPPQI). Conversely, individuals with high external eating tendencies consumed more energy, had a higher percentage of fat intake, and exhibited lower scores on both the Fat Quality Index (FQI) and the Healthy Eating Index (HEI). Those with high scores in restrained eating consumed less energy but showed a higher percentage of protein intake and elevated scores in the Carbohydrate Quality Index (CQI), FQI, Macronutrient Quality Index (MQI), and Healthy Eating Index (HEI). The study concluded that external eating was associated with the least favorable relationship with diet quantity and quality among all eating behaviors, while restrained eating demonstrated the most positive associations, particularly 2 to 4 years after SG surgery (25).

### 3.6. Safety and Complications

In parallel with the rising popularity of BS among patients and clinicians, concerns regarding the safety and complications associated with this surgical procedure have increased. To address these concerns, the TOTS was designed to explore the safety and complications arising from different BS techniques. In this context, 17 studies have been conducted among TOTS participants to evaluate and compare various BS techniques in terms of safety and related complications.

Of the 7,355 patients who underwent BS throughout TOTS, two patients died due to surgery-related complications during the 13-year follow-up period. One patient in the OAGB-200 group developed profound liver failure, severe hypoalbuminemia, and pancytopenia, eventually succumbing a few days after revisional surgery (26). The other patient in the OAGB-160 group developed postoperative peritonitis and underwent urgent peritoneal lavage and antimicrobial therapy. However, the patient passed away 28 days postoperatively following ventilator-associated pneumonia caused by Pseudomonas aeruginosa (27).

The American Society of Metabolic and Bariatric Surgery (ASMBS) defines short-term complications as those occurring within the first 30 days after surgery, while complications beyond this period are categorized as long-term. Major complications are defined as those requiring re-intervention or re-operation, prolonged hospital stays exceeding seven days, or the need for anticoagulant administration (28). 

A prospective study from TOTS, adhering to ASMBS guidelines, demonstrated that both short-term and long-term complications were more pronounced among participants who underwent GB compared to those who underwent SG (P < 0.001) (3). However, due to the limitations of the ASMBS outcome reporting guidelines, many prefer the Clavien-Dindo classification system for surgical complications. Introduced in 2015, this classification provides a more standardized and consistent framework for assessing surgical complications, categorizing them into five grades, ranging from minor deviations from the natural postoperative course to patient death (29, 30).

In a matched control study with approximately three years of follow-up, the Clavien-Dindo classification was used to evaluate post-bariatric complications. This study found no significant difference in early or late complications between SG and GB (31). Additionally, a study focusing on super obese participants (BMI ≥ 50 kg/m²) revealed that post-surgical complications were more prevalent in the OAGB group compared to the SG group, regardless of the biliopancreatic limb (BPL) length (7).

Another prospective study investigated the incidence of cholelithiasis in participants who received prophylactic ursodeoxycholic acid (UDCA) for six months following BS. The cumulative incidence of developing cholelithiasis was 11%, with six-month BMI loss identified as the primary predictor of this condition (32).

There are several indications for revisional surgery following BS, such as insufficient weight loss, weight regain, or post-surgical complications. To date, four investigations within TOTS have focused on revisional surgery, its etiologies, and outcomes. A retrospective study with eight years of follow-up found that out of 5382 primary surgeries, 37 patients required additional surgeries due to complications. A higher proportion of OAGB participants required additional surgeries, mainly due to protein-calorie malnutrition (PCM), while SG participants primarily underwent revisional surgery for gastroesophageal reflux disease (P = 0.021) (33).

Since 2013, the inception year of TOTS, OAGB surgeries were predominantly performed with a 200-cm biliopancreatic limb (BPL). However, in 2016, a case of severe and fatal PCM following OAGB with a 200-cm BPL was reported (26). This prompted further investigations into the unfavorable outcomes associated with OAGB using a 200-cm BPL. For instance, a case series study including seven TOTS participants highlighted severe steatohepatitis in one patient, along with revisional surgeries for PCM in the others following OAGB (27). 

Additionally, another study with one year of follow-up compared OAGB with 160-cm and 200-cm BPLs. While both methods were effective, the 160-cm BPL approach demonstrated superior safety outcomes (34). Consequently, OAGB with a 160-cm BPL became the preferred method for future OAGB surgeries within TOTS.

The rising rates of obesity across all age groups, coupled with the effective weight loss achieved through bariatric surgery, have raised concerns about its safety and efficacy in various age groups and genders. In this context, two studies conducted within the TOTS population focusing on adolescents and older adults demonstrated successful weight loss and significant health improvements following bariatric surgery (8, 9). Additionally, a comparative study involving 707 men and 707 women who underwent bariatric surgery found no significant differences in the effectiveness of the procedures or the severity of complications between genders after 36 months of follow-up (10).

During the coronavirus disease 19 (COVID-19) pandemic, healthcare providers faced significant challenges in prioritizing patient care while accommodating the influx of infected patients. Surgeons were under pressure to postpone most elective procedures, including bariatric surgeries, due to the increased risk of severe COVID-19 complications in patients with diabetes or severe obesity (35-37). However, as the pandemic’s end remained uncertain and delays in bariatric surgeries were associated with poor outcomes, surgical procedures gradually resumed. Debate persists regarding the safety and efficacy of bariatric surgeries performed during the pandemic (38, 39). Researchers within the TOTS study compared 982 participants who underwent surgery prior to the pandemic (March 2017 to March 2018) with 602 participants who underwent surgery during the pandemic (March 2020 to March 2021). The study revealed that both groups achieved comparable 1-year weight loss outcomes and safety profiles, as measured by the Clavien-Dindo classification (40).

## 4. Conclusions

In this review, we examined the TOTS-based studies that have investigated various aspects of BS over the last decade. The topics addressed by the reviewed studies provided valuable insights into the effects of different types of BS on weight loss and its components, risk factors associated with WR, nutritional challenges faced by individuals undergoing BS, and the safety and complications following different BS techniques. Through a series of 36 studies, ranging from preoperative assessments to long-term follow-ups post-surgery, significant findings have emerged at the national level, highlighting the most challenging issues in evaluating the outcomes, effectiveness, and complications of bariatric procedures, as well as the importance of comprehensive nutritional management in this population.

The findings from the TOTS study provide an important overview of the factors associated with the effectiveness of weight loss across different types of BS. They also offer specific insights into several important subgroups, such as metabolically healthy severely obese (MHSO) individuals, severely obese individuals, older adults, adolescents, gender differences, and patients with CKD. Our results underscore the significance of weight components (fat mass and fat-free mass) and emphasize the importance of comorbidity remission in addition to overall weight loss.

In addition, the findings from the TOTS provide crucial insights into the nutritional challenges and considerations surrounding BS. From preoperative assessments to long-term dietary patterns, these studies emphasize the importance of comprehensive nutritional management to optimize patient outcomes and promote long-term health and well-being in individuals undergoing BS. They highlight the need for close follow-up and timely nutritional and therapeutic interventions, which should be systematically and pre-programmed in excellent care centers.

Furthermore, over more than 10 years of follow-up, several studies from TOTS aimed to extend the existing evidence regarding the safety of BS, its different techniques, and post-BS complications from various critical perspectives. Collectively, the results consistently demonstrated the safety of BS across different groups of candidates, including adolescents and older adults, irrespective of sex.

Although TOTS is one of the first prospective cohort studies in the MENA region based on disease registries and examines the safety and efficacy of BS from the perspectives of surgery, medicine, and nutrition in a long-term follow-up, it has certain limitations. As the follow-up period increased, the proportion of missing data also grew. Additionally, the study's design was observational rather than a clinical trial. Moreover, outcomes related to cardiovascular, respiratory, and reproductive systems have not yet been investigated.

Finally, while further cohort studies with longer follow-up durations and larger, more diverse populations are necessary for a comprehensive conclusion, evidence from TOTS studies has highlighted the favorable impact of BS on obesity and related complications, despite its rare major complications.

## Data Availability

The dataset presented in the study is available on request from the corresponding author during submission or after publication.
